# Treatment of massive irreparable rotator cuff tears through biodegradable subacromial InSpace Balloon

**DOI:** 10.1186/1471-2482-13-S1-A43

**Published:** 2013-09-16

**Authors:** Donato Rosa, Giovanni Balato, Giovanni Ciaramella, Sigismindo Di Donato, Nicola Auletta, Claudia Andolfi

**Affiliations:** 1 Department of Orthopaedic Surgery, Federico II University, Naples, Italy

## Introduction

The massive irreparable tears of rotator cuff represents a debilitating condition for the patient as well as it is still an hard challenge to an orthopedic surgeon [1,2]. Treating these tears is an interesting and evolving issue due to the development of arthroscopic surgery and of the materials used for treatment. There are several surgical options for treating massive tears, among these the *arthroscopic debridement* and the *implant of a reverse prosthesis *[3]. Recently, the Biodegradable InSpace Balloon device has been put on the market, once implanted between the acromion and the proximal humeral epiphysis, this device guarantees significant pain reduction and range of motion (ROM) improvement in patients affected by massive rotator-cuff shoulder tears, as it restores the correct expanse of the pathologically reduced subacromial space.

Primary end-points include: pain relief, improvement in the range of motion, activities of daily living, and enhancement of shoulder strength referring to the Constant Score [5] recorded at each follow-up visit.

## Materials and methods

Since September 2012 we have implanted the Biodegradable InSpace Balloon in 10 patients (3 females and 7 males; average age 69 years) affected by an irreparable massive rotator-cuff tear. All patients were preoperatively assessed by physical examination, RX and RMN examinations. Prospective postoperative assessment of symptoms and complications and/or device-related adverse events were recorded with prospective determination of the Constant Score as well as at hospital discharge, 1, 3 and 6 weeks, 3 and 6 months postoperatively. All operations were performed with the patient in a beach-chair position under general anesthesia. This device has been inserted arthroscopically, using 3 arthroscopic ports (anterior, lateral and posterior or posterior-lateral) in the subacromial space. After subtotal removal of the subacromial bursa, the tear was debrided and the rotator cuff was assessed by grasping the edges of the tendons with an arthroscopic clamp, in an attempt to draw it to the footprint region. A decision was made to insert the balloon when it was deemed that the rotator cuff tear was irreparable. During the postoperative period the patients have followed a rehabilitation program which consisted in immobilizing the arm with a 4 week brace and, after that, in an active/passive motor physical therapy. The patients were taught simple motor exercises to do at home in order to hasten the rehabilitation process and were advised avoiding heavy activities during the first three months after the surgery.

## Results

There was a significant reduction of pain score and night pain one week following balloon implantation. This improvement increased progressively throughout the whole duration of follow-up. Patients reported significant improvement in their daily living activities the sixth week after surgery. The range of motion also showed significant improvement 8 weeks later the surgery.

In all measurable parameters, once reached significance, the improvement was maintained throughout the follow-up period. All patients were satisfied with the results and showed significant functional improvements just a few time after the surgery. No postoperative complications showed up.

## Conclusion

This study has shown efficacy and a very high clinical safety profile of the InSpaceTM device in a small group of patients with massive irreparable rotator cuff tears. The insertion of the device was associated with significant early improvement in subjective pain scores and a decrease in reported night pain. The total Constant Score showed statistically significant improvement as did scores of activities of daily living and range of motion. It’s a simple non-invasive surgery, technically esay to replicate with preliminary encouraging results. This procedure represents a valid surgical option in order to postpone more invasive operations.
